# Clinical assessment of the physical activity pattern of chronic fatigue syndrome patients: a validation of three methods

**DOI:** 10.1186/1477-7525-7-29

**Published:** 2009-04-01

**Authors:** Korine Scheeres, Hans Knoop, van der Jos Meer, Gijs Bleijenberg

**Affiliations:** 1 Expert Centre Chronic Fatigue, Radboud University Nijmegen Medical Centre (4628), PO Box 9101, 6500 HB Nijmegen, The Netherlands; 2 Department of General Internal Medicine, Radboud University Nijmegen Medical Centre, Nijmegen, The Netherlands

## Abstract

**Background:**

Effective treatment of chronic fatigue syndrome (CFS) with cognitive behavioural therapy (CBT) relies on a correct classification of so called 'fluctuating active' versus 'passive' patients. For successful treatment with CBT is it especially important to recognise the passive patients and give them a tailored treatment protocol. In the present study it was evaluated whether CFS patient's physical activity pattern can be assessed most accurately with the 'Activity Pattern Interview' (API), the International Physical Activity Questionnaire (IPAQ) or the CFS-Activity Questionnaire (CFS-AQ).

**Methods:**

The three instruments were validated compared to actometers. Actometers are until now the best and most objective instrument to measure physical activity, but they are too expensive and time consuming for most clinical practice settings. In total 226 CFS patients enrolled for CBT therapy answered the API at intake and filled in the two questionnaires. Directly after intake they wore the actometer for two weeks. Based on receiver operating characteristic (ROC) curves the validity of the three methods were assessed and compared.

**Results:**

Both the API and the two questionnaires had an acceptable validity (0.64 to 0.71). None of the three instruments was significantly better than the others. The proportion of false predictions was rather high for all three instrument. The IPAQ had the highest proportion of correct passive predictions (sensitivity 70.1%).

**Conclusion:**

The validity of all three instruments appeared to be fair, and all showed rather high proportions of false classifications. Hence in fact none of the tested instruments could really be called satisfactory. Because the IPAQ showed to be the best in correctly predicting 'passive' CFS patients, which is most essentially related to treatment results, it was concluded that the IPAQ is the preferable alternative for an actometer when treating CFS patients in clinical practice.

## Introduction

Chronic fatigue syndrome (CFS) is characterized by unexplained severe fatigue that does not resolve with bed rest, lasts for at least six months and causes serious reductions in daily functioning [1]. Spontaneous recovery rates are low [2,3]. Reviews of randomized controlled trials (RCTs) have shown that cognitive behavior therapy (CBT) is the most effective treatment for CFS [4-6]. In one of the RCTs included in these reviews it was found that CFS patients' individual level of daily physical activity predicted the CBT treatment outcome (Prins et al., 2001) [7]. Based on their daily activity level, CFS patients can be divided into two subgroups, distinguishing 'fluctuating active' from 'passive' CFS patients in a proportion of 75% versus 25% respectively [8]. Fluctuating active patients generally show infrequent bursts of activity followed by extreme exhaustion, whereas passive patients usually avoid activities as much as possible. In the trial of Prins et al. [7] it turned out that passive CFS patients showed almost no improvement. It was suggested that these passive patients might need a different type of treatment. Therefore a more appropriate protocol was developed [9] and tested [10]. It is especially important that passive patients are being recognized correctly, as they do not recover when they receive the protocol for active patients [7,11]. When active patients accidentally receive the passive protocol, the consequences are less problematic. They start complaining quickly of exhaustion when increasing their already fluctuating activity pattern; this protest alerts the therapists and enables them to adjust the protocol so that recovery can still be reached.

One of the major innovations of the new protocol is that whereas fluctuating active patients start with practicing a base line activity level that prevents bursts of activity, passive patients start directly with increasing physical activity [11,12]. The adapted protocol has proven to be as effective for passive CFS patients as the original protocol is for pervasively active CFS patients [10]. Therefore it is now being used in clinical practice for passive CFS patients.

Now in order to decide which protocol should be used, it is necessary to assess the patient's physical activity pattern. An accurate and objective method to do this is using an actometer. Actometers are based on an electric sensor and yield highly reliable data [8,13,14]. But for clinical settings actometers are not feasible because of a lack of personnel to instruct patients how to wear the actometer and to read out the computerized graphics. Besides, actometers can be rather expensive. An alternative method is the short 'Activity Pattern Interview' (API) of daily activities [9,12]. Talking through a typical day by asking concrete questions about activities 'from minute to minute' should deliver the needed information. Although this method is now being used in several settings and is also included in trainings of CBT for CFS, its validity has never been tested.

Another possibility is using self-report questionnaires. Since the outcome of such a questionnaire is not dependent on the therapist's skills, the validity of such a questionnaire might be higher and more stable than that of an interview. Several 'physical activity questionnaires' are available for this purpose [15-18]. In this study the widely used and validated International Physical Activity Questionnaire (IPAQ) [18], and a newly developed CFS Activity Questionnaire (CFS-AQ) were evaluated. We developed the CFS-AQ because it was presumed that the IPAQ, as other existing activity questionnaires designed for the general population, might not suit the typical low activity levels of CFS patients.

The present study evaluates and validates these three alternative methods to assess CFS patients activity pattern. The research questions of the present study are: 1. What is the validity and sensitivity of the API when assessing CFS patients' activity pattern? 2. Do the IPAQ and the CFS-AQ assess activity pattern better than the API? The hypothesis was that the CFS-AQ would show a higher validity than the IPAQ and the API.

## Methods

### Subjects and procedure

In this study 226 consecutive CFS-patients aged between 16 and 65 participated. They were all referred between January 2004 and October 2005 by a medical specialist or general practitioner to the Nijmegen Expert Centre for Chronic Fatigue (ECCF). All participants fulfilled the CDC-94 criteria for CFS [1]. The main complaint of severe fatigue was indicated by scores of 35 or higher on the Checklist Individual Strength (CIS) subscale 'fatigue severity' [19]. Severe impairment was defined by a cut off score of 700 or higher on the Sickness Impact Profile (SIP) [20]. Data for this study were obtained during the patients first two visits to the centre. During the intake session the six therapists participating in this study performed the API. All therapists were trained and experienced in CBT for CFS. Their training included assessing the activity pattern of the patient with the API. During the second visit (diagnostic test session) patients completed the IPAQ and the CFS-AQ. Additionally they got instructions to wear the actometer for the next two weeks. Before starting this study it was judged by the Nijmegen Medical Hospital Ethical Commission, who indicated no need for informed consent.

### Measures

#### API

The API investigates the usual activities of a patient on a typical day. It consists of a form with three relevant topics that are questioned during the intake interview. On bases of these topics the therapist forms a final judgement in the form of a dichotomous outcome, namely 'passive' or 'active'. These topics are: the routine pattern of activities and the amount of time laying or sitting during the day of yesterday, the number of times leaving the house during a day and practicing a paid or unpaid job or not. To investigate the routine daily living pattern, the day of yesterday is being questioned 'from minute to minute', as concrete as possible, like it would be registered by a camera. To minimise a recall-bias, usually the day of yesterday is being used, only when this was not a typical day or a weekend day another day in the past week is being investigated.

The passive CFS patient can be recognized by spending a great deal of time lying down, going out infrequently and not practicing a job anymore. In most patients it takes about ten minutes to administer the API. The therapists were instructed to fill in this form during the intake, their definite activity pattern judgement included.

#### IPAQ

The short form IPAQ is a 9-item scale, providing information on the amount of minutes spent in vigorous and moderate intense activity and walking during the last 7 days, for which separate sub scale scores can be calculated, for work-related, transportation, housework/gardening and leisure-time activity. Filling in the IPAQ takes about 5–7 minutes. The IPAQ has a good test-retest reliability (Spearman's ρ = 0.80) and a moderate criterion validity (Spearman's ρ = 0.30) with an accelerometer [18].

#### CFS-AQ

The activity questionnaire was developed at the ECCF. It contains 10 items concerning questions about activities in the last two weeks, with four sub scales: 'physical activity' (four questions), 'rest' (four questions), 'using aids' (one question) and 'social activity' (one question). Each item is scored on a four point Likert scale. It takes about 5–7 minutes to fill in the CFS-AQ. The CFS-AQ internal consistent reliability and test-retest reliability were tested in this study's population and were acceptable (Cronbach's alpha 0.73; Spearman's ρ 0.72).

#### Actometer

The actometer (^©^Actilog V3.0) is a little box (43*29*16 mm) that has a piëzo-electric sensor, which is sensitive in three directions. It is worn on the ankle, usually for 14 days in order to retain 12 complete registration days. Sensor acceleration results in an output signal, all signals above a certain threshold are added to a pulse generator. This pulse generator triggers a 2-bit counter that adds the registered value to an 'integration counter' every five minutes [13], from which mean Daily Physical Activity scores are computed. Actometers yield valid and highly reliable data in the healthy population [13] and in the CFS population [8,14].

The activity pattern of passive CFS patients is defined by scoring zero or one of the 12 days above a CFS reference score. This reference score is the mean Daily Physical Activity score of CFS patients. Fluctuating active CFS patients score two or more of the 12 days above this CFS reference score and mostly present an irregular, 'fluctuating' graphic.

### Analyses

First, correlations between the three instruments and the continuous actometer scores were analyzed. After that logistic regression analyses were performed with the IPAQ and the CFS-AQ, using actometer typology as dependent variable, to gain their predicted probability scores and a dichotomous outcome scale of activity level. From the regression analysis results we obtained the parameters for the CFS-AQ and the IPAQ that are needed to prescribe a formula that can predict a patients' activity pattern according to the actometer. The formula for the CFS-AQ predicting the probability that a particular patient is active became:



('physical' = score on subscale 'physical activity', 'rest'= score on subscale 'rest', 'aids' = score on subscale 'using aids', and 'social' = score on subscale 'social activities').

And the formula for the IPAQ predicting the probability that a particular patient is active became:



('walking'= score on subscale 'walking', 'moderate'= score on subscale 'moderate activities', 'heavy'= score on subscale 'heavy activities'). The API did not need such analysis, since it resulted in a dichotomous outcome directly.

**Figure 1:**
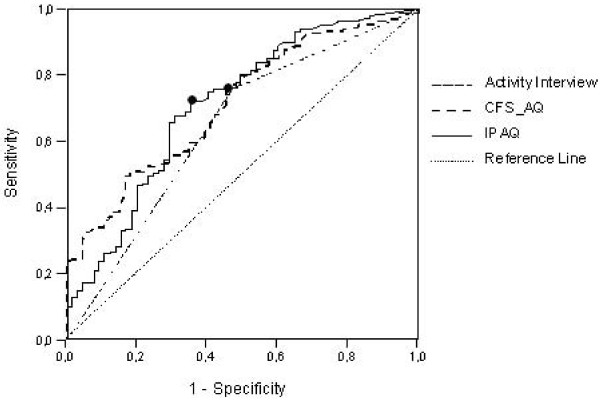
**Receiver operating characteristic (ROC) curves of the IPAQ, CFS-AQ and API predicting CFS-patients daily activity typology, best cut-off points of CFS-AQ and IPAQ marked with (black circle)**.

With the predicted probability scores derived from the regression analysis, receiver operating characteristic (ROC) curves were constructed for all three instruments in order to analyze sensitivity and specificity levels (figure 1). 'Sensitivity' represented the proportion of passive patients correctly classified as passive, whereas 'specificity' was defined as the proportion of active patients correctly classified as active. A ROC curve shows the trade-off between sensitivity and specificity for all possible predicted values. The ROC area under the curve represents the validity of a model. The higher the curve and the more it follows the vertical axis, the more accurate the model [21]. An area of 1 represents a perfect validity whereas an area of 0,5 would be identical to just guessing. A rough guide for classifying the accuracy of a diagnostic test is the traditional academic point system: .90–1 = excellent, .80–.90 = good, .70–.80 = fair, .60–.70 = poor, .50–.60 = fail.

For each curve, at one 'cut off' point the combination of sensitivity and specificity is optimal [21]. The best cut off points for the IPAQ and the CFS-AQ were determined by calculating and computing sensitivity and specificity at different cut off points and find the point with the highest score. Cross tabulations were constructed to support comparison of the three methods. Finally it was investigated whether the areas under ROC curves were significantly different by calculating a Z score using the formula of Hanley & Mc Neil [22]. All statistical analyses were conducted using SPSS version 12.01.

## Results

### Descriptives and demographics

The mean age of participating patients was 37 years (SD 11.3 range 15–68). The male/female ratio was 26%/74% (59 male, 167 female), median duration of fatigue was 5 years (range 2–32). According to the actometer measures, 29% of the patient population had a passive activity pattern and 71% had a fluctuating active one.

### Validity of the instruments

The correlation of the API and the IPAQ with the actometer scores appeared to be weak (Spearman's ρ = 0.27 and ρ = 0.33 respectively). The CFS-AQ showed a moderate correlation with the actometer (Spearman's ρ = 0.41).

Figure 1 and table 1 show the area under the curve (representing the validity) for the CFS-AQ, the IPAQ and the API. As can be seen, the validity of the API (0.643) was somewhat smaller than that of the two questionnaires (0.710 and 0.711). Following the method of Hanley and Mc Neil [22], the validity of the three instruments was not significantly different however [see additional file 1].

**Table 1:** Area under the ROC curve of the API, CFS-AQ and the IPAQ

Test Results Variables	Area Under the Curve	Std. Error	Asymptotic significance ^b^	Asymptotic 95% confidence Interval	
				
				Lower bound	Upper bound
Activity Pattern Interview	0.643	0.042	0.001	0.562	0.725
CFS Activity Questionnaire	0.710	0.036	0.000	0.640	0.781
IPAQ	0.711	0.039	0.000	0.634	0.788

### Sensitivity of the instruments

Based on the API, 52.3% of all passive CFS patients were correctly classified as passive (sensitivity) and 75.8% of all active patients were correctly classified as fluctuating active (specificity) (table 2). The optimum predicted probability cut off for the CFS-AQ was at 0.73, by which a sensitivity of 64.6% was reached combined with a specificity of 65.2% (table 3). For the IPAQ the best predicted probability cut off was at 0.67 with a sensitivity of 70.1% and a specificity of 62.7% (table 4).

**Table 2:** Sensitivity and specificity of the Activity Pattern Interview (N = 226)

		**Actometer typology**		
		Passive	Active	
**Activity Pattern Interview**	Passive (N/%)	34/(52.3%) (= sensitivity)	39/(24.2%)	73 (32%)
	Active (N/%)	31/(47.7%)	122/(75.8%) (= specificity)	153 (68%)
		65/(100%)	161/(100%)	226 (100%)

Predictive value of a positive test (sensitivity) PV+ = 34/65 = 0.523

Predictive value of a negative test (specificity) PV- = 122/161 = 0.758

**Table 3:** Sensitivity and specificity of the CFS-AQ with the optimum cut off point at 0.73 (N = 226)

		**Actometer typology**		
		Passive	Active	
**CFS-AQ**	Passive (N/%)	42/(64.6%)	56/(34.8%)	98 (43.4%)
	Active (N/%)	23/(35.4%)	105/(65.2%)	128 (56.6%)
	65/(100%)	161/(100%)	226/(100%)	

Predictive value of a positive test (sensitivity) PV+ = 42/65 = 0.646

Predictive value of a negative test (specificity) PV- = 105/161 = 0.655

**Table 4:** Sensitivity and specificity of the IPAQ with the optimum cut off point at 0.67 (N = 226)

		**Actometer typology**		
		Passive	Active	
**IPAQ**	Passive (N/%)	46/(70.1%)	60/(37.3%)	106 (47%)
	Active (N/%)	19/(29.3%)	101/(62.7%)	120 (53%)
		65/(100%)	161/(100%)	226 (100%)

Predictive value of a positive test (sensitivity) PV+ = 46/65 = 0.701

Predictive value of a negative test (specificity) PV- = 101/161 = 0.627

For scoring the IPAQ and the CFS-AQ, the formula's given in this article have to be filled in the following way: the scores on the different subscales of a particular patient have to be filled in at the corresponding place in the formula. Afterwards the formula has to be calculated, which can best be done by using a program like Excel. Finally one should check whether the outcome of this calculation lies above or below the cut off point of 0.73 (when scoring the CFS-AQ) or 0.67 (when scoring the IPAQ).

## Discussion

This study investigated whether the structured 'Activity Pattern Interview' (API), the International Physical Activity Questionnaire (IPAQ) and the CFS-Activity Questionnaire could accurately assess the daily activity pattern of CFS patients.

It appeared that all three instruments had a fair validity and none of the instrument was significantly better than the others. Contrary to our hypothesis, the CFS-AQ, a questionnaire specifically developed for CFS, was not more accurate than the IPAQ or the API. This implies in the first place that the design of a questionnaire especially for CFS patients did not result in a significantly higher validity than the already existing IPAQ. Secondly, these results show that some training and experience in performing the API interview are apparently enough to almost equal a questionnaire. A third implication, of the finding that all three instruments showed a fair validity, is that the three tested instruments could all be used to predict activity pattern in CFS patients, but that the rather high proportion of false predictions remains a serious problem that needs attention in future studies.

A practical question that remains is: what should be advised for therapists in clinical practice? Which of the three instruments could best be used if no actometers are available? Although the validity of the three instruments appeared to be fair, and hence in fact none of the instruments can really be called satisfactory, the percentage of correctly classified passive patients (sensitivity) was the highest for the IPAQ (namely 70.1%, table 4). Given the fact that especially the unjust classification of passive patients as active should be minimized, since they do not recover with the protocol for active patients [11], we advise that the IPAQ is now the best available alternative for an actometer.

An advantage of the IPAQ is that its original scoring protocol provides a categorical outcome of three activity levels (low, moderate and high) and even a continuous score (calculating MET per minutes). Although these algorithms are not useful for the purpose of classification these outcomes might provide useful information for counseling sessions or follow-up information.

From a more practical perspective one could argue to use the API instead of the IPAQ, since it has a direct dichotomous outcome and hence does not need the use of complicated formulas. However, since adequate use of the API asks for therapists who are experienced with CFS and trained in using the interview, this advise should not be given to therapists without such experience.

A strong point of this study is the fact that it concerns a clinically relevant question with important treatment consequences. A methodological limitation of this study is the fact that the predicted models of the questionnaires were derived from the same population as in which afterwards the ROC curves were constructed. This implies that, when tested in a possible validation study, the instruments might show a somewhat lower validity, although probable still better than 'just guessing'.

Compared to other studies, the correlations of the three tested instruments with objective measurements are not different from other established self-report physical activity questionnaires. None of the regularly used physical activity questionnaires that have been validated against objective measurements, e.g. the SQUASH [18] the LASA [17] or the Baecke questionnaires [16] have shown correlations above 0.45. A review that summarized reliability and criterion validity for seven questionnaires, reported a median validity correlation of about 0.3 [23]. All self report measurements of physical activity seem to suffer from the inherent problem that people are not able to report this kind of behavioral aspects correctly. Besides that, accidental activity peaks or rest periods might influence actometers more than questionnaire results, which might also limit correlations. Probably more benefits can be gained with a daily registration by the patient of physical activity. Another option could be to include the opinion of a direct partner of the patients in the reporting of daily activities.

## Conclusion

To conclude, the results of this study suggest that mearusing physical activity pattern in CFS patients with self-report measurements remains a difficult matter. The contributions of this study are in the first place the finding that a specific 'CFS activity questionnaire' does not result in higher validity than that of already existing questionnaires. Secondly, this study makes clear that the validity of an 'Activity Pattern Interview' for CFS patients is not directly better than that of self report questionnaires.

For the clinical practice of CFS treatments, this study's results indicate that, when actometers are not available, the IPAQ is the preferred alternative instrument to use, because of its somewhat lower level of unjust passive classifications.

## Competing interests

The authors declare that they have no competing interests.

## Authors' contributions

KS collected all data and built up the database, designed and performed the statistical analysis and wrote the manuscript. HK contributed to the development of the study design and advised about the performance of the statistical analysis. JM contributed to the selection of patients. The analysis and results were discussed with the three authors together. HK, JM and GB revised the manuscript critically several times. GB delivered the treatment outcome measurement scales, helped with interpreting the results and helped to draft the manuscript. All authors read and approved the final manuscript.

## Supplementary Material

Additional file 1 **Calculation of the significance level (Z score) of the difference between the area's under the ROC curves between the IPAQ, the CFS-AQ and the API.** The data provide the formula's used to calculate the significance level (Z score) of the difference between the area's under the ROC curves between the IPAQ, the CFS-AQ and the API. Click here for file
