# Supplementary figures and images for: Clinical assessment of the physical activity pattern of chronic fatigue syndrome patients: a validation of three methods
Source: Health Qual Life Outcomes. 2009 Apr 1;7:29. doi: 10.1186/1477-7525-7-29 (PMC2674446; doi:10.1186/1477-7525-7-29)

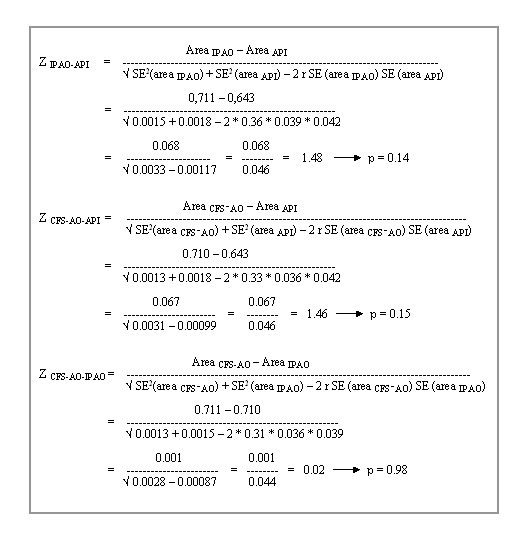

Supplement: Additional file 1 — Calculation of the significance level (Z score) of the difference between the area's under the ROC curves between the IPAQ, the CFS-AQ and the API. The data provide the formula's used to calculate the significance level (Z score) of the difference between the area's under the ROC curves between the IPAQ, the CFS-AQ and the API. [file 1477-7525-7-29-S1.jpeg]
